# Prognostic Impact of Metastatic Pattern in Renal Cell Carcinoma: IMDC, IMDC-7, and Meet-URO Scores

**DOI:** 10.15586/jkc.v13i2.456

**Published:** 2026-06-22

**Authors:** Okan Cetin, Nihan Eren, Melin Aydan Ahmed, Meltem Ekenel, Mert Basaran

**Affiliations:** 1 Department of Medicine, Istanbul University School of Medicine, Istanbul, Turkey;; 2 Department of Medical Oncology, Istanbul University Institute of Oncology, Istanbul, Turkey

**Keywords:** IMDC, IMDC-7, Meet-URO, metastatic renal cell carcinoma, prognosis, risk score

## Abstract

The International Metastatic Renal Cell Carcinoma Database Consortium (IMDC) score is widely used for prognostic stratification in metastatic renal cell carcinoma (mRCC); however, the value of extended models incorporating metastatic site information remains uncertain in patients treated with first-line tyrosine kinase inhibitor (TKI) monotherapy. We retrospectively analyzed 183 patients with mRCC who received first-line TKI therapy (sunitinib, pazopanib, or cabozantinib) between 2013 and 2023. The overall survival (OS) and progression-free survival (PFS) were assessed using Kaplan–Meier analysis and Cox regression models, and the prognostic performance of IMDC, IMDC-7, and Meet-URO scores was compared using Harrell’s concordance index. At baseline, 47% of patients had metastases to high-risk sites (bone, liver, or brain), and 36% had three or more metastatic sites. In univariate analyses, bone, liver, and brain metastases as well as the presence of ≥3 metastatic sites were associated with significantly shorter OS and PFS. On multivariable analysis, anemia, poor performance status, absence of prior nephrectomy, and ≥3 metastatic sites independently predicted worse OS, while anemia, poor performance status, and bone metastasis remained independently associated with inferior PFS. All three prognostic models effectively stratified survival outcomes; however, IMDC-7 demonstrated the highest discriminatory ability for OS, followed by IMDC and Meet-URO. These findings highlight the strong prognostic impact of both metastatic burden and distribution in mRCC and support the incorporation of metastatic site assessment into routine risk stratification, particularly in settings where access to immune-based combination therapies remains limited.

## Introduction

Renal cell carcinoma (RCC), the most common kidney cancer in adults, accounts for approximately 2.2% of all cancers worldwide and ranks 14th in global cancer incidence (1). Once the RCC progresses to a metastatic stage, prognosis declines substantially. However, even among patients with metastatic disease, prognosis can vary widely. This heterogeneity highlights the ongoing need for a reliable, practical, and low-cost prognostic risk score.

The International Metastatic RCC Database Consortium (IMDC) score is one of the most widely used risk scores in metastatic RCC (mRCC). Additional variables, such as metastatic site distribution and systemic inflammation markers, have been identified as significant predictors of prognosis (2–6). The IMDC-7 and Meet-URO scores have been developed by incorporating these variables into the IMDC. The Meet-URO score, which combines the IMDC score with the neutrophil-to-lymphocyte ratio (NLR) and bone metastases, was initially developed in patients receiving immune checkpoint inhibitors (ICIs) and categorizes patients into five prognostic groups (7, 8). More recently, studies in patients treated with tyrosine kinase inhibitor (TKI) monotherapy or TKI–ICIs combinations have used the new version of the Meet-URO score, which categorizes patients into three prognostic groups (9, 10). The IMDC-7 score, in turn, which adds the presence of brain, bone, and/or liver metastases as the initial site of systemic disease to the original IMDC score, has been externally validated (11, 12).

Importantly, the prognostic performance of these models may vary depending on the therapeutic context in which they are applied. Over the past decades, mRCC treatment strategies have evolved, transitioning from cytokine-based therapies to targeted agents, and more recently to immune checkpoint inhibitors. Although immunotherapy is now a cornerstone in the treatment of mRCC, targeted therapies, such as TKIs, remain widely used, especially in patients who are not eligible for immunotherapy and in many low- and middle-income countries where access is limited. Moreover, current clinical guidelines continue to recommend first-line TKI therapy for selected patients with favorable-risk disease (13, 14).

In this study, we aim to evaluate the prognostic impact of metastatic sites in mRCC patients treated with first-line TKIs. We also compare the prognostic performance of extended risk scores that incorporate these factors, such as IMDC-7 and Meet-URO, to the original IMDC score in our cohort to determine which performs best. Given that prior validations of these models have primarily involved ICI-based regimens or later-line TKIs, their prognostic value in patients receiving first-line TKI monotherapy has not been systematically assessed and therefore represents the central focus of our investigation.

## Materials and Methods

### Study design

We conducted a retrospective study of patients with mRCC treated at Istanbul University Oncology Institute between 2013 and 2023. All patients had histologically confirmed RCC (clear-cell or non–clear-cell) and received first-line TKI. Sunitinib was administered at 50 mg daily on a 4-weeks-on/2-weeks-off schedule, with dose reductions to 37.5 and 25 mg as needed. Pazopanib was initiated at 800 mg once daily with stepwise reductions to 400 and 200 mg. Cabozantinib was started at 60 mg once daily, with permitted reductions to 40 and 20 mg. Patients younger than 18 or older than 85 years, or with a second primary malignancy or incomplete clinical and laboratory data, were excluded from the study. Collected variables included demographic characteristics, tumor pathology and staging, treatment details, pretreatment laboratory parameters, and survival outcomes. The study was conducted in accordance with the guidelines of the Declaration of Helsinki and received ethical approval from the Istanbul University Ethics Committee (Approval No. 3283077, dated March 7, 2025).

### Risk scores

IMDC, IMDC-7, and Meet-URO scores were calculated for each patient. The IMDC score is composed of six variables: Karnofsky performance status <80, time from diagnosis to treatment <1 year, hemoglobin <12 g/dL, corrected calcium >10.2 mg/dL, neutrophils >7000/µL, and platelets >400,000/µL. Patients are classified as favorable (0), intermediate (1–2), or poor risk (≥3 points). The IMDC-7 score extends this by adding the presence of brain, bone, or liver metastases as a seventh risk factor; the scoring structure is shown in Table 1 (12). The Meet-URO score integrates NLR and the presence of bone metastases into the IMDC score; the scoring structure is shown in Table 2 (9).

**Table 1:** IMDC-7 risk score assessment.

IMDC-7 risk score components	IMDC-7 risk score classification
0 positive risk factors	Favorable risk
1–2 positive risk factors	Intermediate risk
≥3 positive risk factors	Poor risk
IMDC-7 risk factors include: • Karnofsky performance status <80% • Time from diagnosis to treatment <1 year • Hemoglobin below the lower limit of normal • Corrected calcium above the upper limit of normal • Neutrophil count above the upper limit of normal • Platelet count above the upper limit of normal • Presence of brain, bone, or liver metastasis	

**Table 2:** Meet-URO risk score assessment.

Meet-URO risk score components	Meet-URO risk score classification
[None] or [Bone metastasis] or [NLR ≥ 3.2] or [IMDC intermediate risk]	Favorable risk
[IMDC intermediate risk + (Bone metastasis and/or NLR ≥ 3.2)] or [IMDC high risk] or [IMDC high risk + (Bone metastasis or NLR ≥ 3.2)]	Intermediate risk
[IMDC poor risk + Bone metastasis + NLR ≥ 3.2]	Poor risk

NLR, neutrophil-to-lymphocyte.

### Statistical analysis

Statistical analyses were performed using IBM SPSS Statistics for Windows, version 28.0 (IBM Corp., Armonk, NY, USA) and Python 3.12. A P-value <0.05 was considered statistically significant. Continuous variables were presented as medians with ranges, while categorical variables as percentages (%) and counts (n). Overall survival (OS) was calculated from the initial date of TKI treatment to the date of death or the last follow-up, while progression-free survival (PFS) was calculated from the initial date of TKI treatment to the date of disease progression, death, or last follow-up. Survival curves were estimated using the Kaplan–Meier method, and group differences were compared using the log-rank test. Univariate and multivariate Cox regression analyses were performed to identify prognostic factors associated with survival. Variables with P < 0.05 in the univariate analysis were included in the multivariate model using forward likelihood ratio selection. Hazard ratios (HRs) and 95% confidence intervals (CIs) were reported. The prognostic performance of the IMDC, IMDC-7, and Meet-URO scores was assessed using Harrell’s concordance index (c-index), calculated with the *concordance_index* function from the Lifelines package in Python 3.12.

## Results

### Patient characteristics

A total of 183 patients with mRCC were included. The median age was 56 years (range, 18–85 years), and 76% (n = 139/183) of patients were male. Half of the patients had de novo metastasis, while the rest had metachronous metastatic disease. The most common metastatic sites at diagnosis of mRCC were the lung (67%, [n = 123/183]), bone (33%, [n = 60/183]), and liver (15%, [n = 28/183]). Overall, 86 (47%) patients had at least one of these most common metastatic sites. Only 13% (n = 23/183) of patients had a Karnofsky performance status <80, indicating they were not independent in daily activities. Baseline characteristics of all patients are summarized in Table 3.

**Table 3:** Baseline characteristics.

Variable	All Patients (N = 183)
Mean age at diagnosis, years (range)	56 (18–85)
Gender, n (%)	
Male	139 (76)
Female	44 (24)
Tumor histopathology,n (%)	
Clear cell	147 (80)
Non-clear cell	25 (14)
Missing	11 (6)
Mean tumor size, cm (range)	8 (2–20)
Tumor histological grade, n (%)	
1–2	33 (18)
3–4	102 (56)
Tumor morphology and invasive features, n (%)	
Necrosis	84 (46)
Sarcomatoid/rhabdoid differentiation	32 (18)
Inferior vena cava invasion	11 (6)
Metastasis type, n (%)	
De novo metastatic	105 (57)
Metachronous metastatic	78 (43)
Number of metastases,n (%)	
<3	118 (64)
≥3	65 (36)
Site of metastasis, n (%)	
Lung	123 (67)
Bone	60 (33)
Liver	28 (15)
Brain	14 (8)
Adrenal	38 (21)
Presence of at least one of bone, brain, or liver metastases, n (%)	86 (47)
Karnofsky performance status, n (%)	
≥80: Independent in daily activities	160 (87)
<80: Not independent in daily activities	23 (13)
Time from diagnosis to systemic therapy, months (range)	22 (0–215)
First-line therapy, n (%)	
Sunitinib	109 (59)
Pazopanib	69 (38)
Cabozantinib	5 (3)
Treatment interventions, n (%)	
Nephrectomy	133 (72)
Radiotherapy	69 (38)
Metastasectomy	34 (19)

### Survival outcomes

At the median follow-up of 15 months, the median OS and PFS were 18 months (95% CI, 12–23) and 9 months (95% CI, 7–10), respectively. OS and PFS were analyzed in relation to metastatic patterns to provide clearer insights. Patients with fewer than three metastatic sites had significantly longer OS (23 vs 12 months, P = 0.003) and PFS (11 vs 7 months, P = 0.005). Metachronous metastases were also associated with better survival compared to de novo disease (OS: 30 vs 13 months, P = 0.007; PFS: 12 vs 8 months, P = 0.083). Among the sites of metastasis, the presence of metastases in the bone, liver, or brain was linked to shorter OS (bone: 12 vs 23 months, P = 0.006; liver: 10 vs 19 months, P = 0.010; brain: 6 vs 19 months, P = 0.006). A similar trend was observed in PFS (bone: 7 vs 10 months, P = 0.001; liver: 6 vs 9 months, P = 0.043; brain: 6 vs 9 months, P = 0.014). Patients with at least one of these three high-risk metastatic sites had significantly shorter OS (12 vs 25 months, P < 0.001) and PFS (7 vs 11 months, P < 0.001). In contrast, lung and adrenal metastases were not found to be significantly associated with differences in survival.

To identify variables associated with OS following the initiation of first-line therapy, we performed univariate Cox regression analyses on several potential prognostic factors. Our results indicated that anemia, neutrophilia, thrombocytosis, hypercalcemia, low Karnofsky Performance Status, a diagnosis-to-treatment interval of less than 1 year, absence of prior nephrectomy, de novo metastatic disease, a greater number of metastatic sites, metastases involving the bone, liver, brain, or adrenal glands, and elevated NLR were significantly associated with worse survival outcomes (Table 4). We then conducted multivariate Cox regression analysis with forward likelihood ratio selection from the full model, and only four independent variables, including anemia, low Karnofsky Performance Status, absence of prior nephrectomy, and a higher number of metastatic sites, were shown to have independent impacts on OS (Table 4).

**Table 4:** Univariate and multivariate analysis for overall survival.

	n	Univariate Analysis			Multivariate Analysis		
		HR	%95 CI	p-value	HR	%95 CI	P
Age at diagnosis				0.358			
≤65	143	1					
>65	40	1.20	0.81–1.76				
Gender				0.911			
Male	139	1					
Female	44	0.97	0.67–1.43				
Tumor histopathology				0.165			
Clear cell	147	1					
Non-clear cell	36	1.33	0.88–2.00				
Hemoglobin *ε*				**<0.001**			**<0.001**
<12 g/dL	81	1			1		
≥12 g/dL	102	0.36	0.25–0.51		0.40	0.26–0.61	
Neutrophilsμ				**0.012**			
≤7000/µL	138	1					
>7000/µL	45	1.66	1.11–2.49				
Plateletsμ				**0.005**			
≤400/10^3^µL	144	1					
>400/10^3^µL	39	1.75	1.18–2.59				
Corrected Calciumμ				**<0.001**			
≤10.2 mg/dL	159	1					
>10.2 mg/dL	24	2.34	1.51–3.64				
Karnofsky Performance Status				**<0.001**			**<0.001**
≥80	160	1			1		
<80	23	15.43	8.54–27.87		12.07	6.08–23.9	
Time from diagnosis to treatment				**0.002**			
<1 year	110	1					
≥1 year	73	0.57	0.40–0.81				
Nephrectomy				**<0.001**			**0.048**
No	50	1			1		
Yes	133	0.35	0.25–0.52		0.64	0.42–0.99	
Metastasis type				**0.008**			
De novo metastatic	105	1					
Metachronous metastatic	78	0.63	0.45–0.88				
Number of metastases				**0.004**			**0.002**
<3	118	1			1		
≥3	65	1.64	1.17–2.30		1.96	1.29–2.98	
Lung metastasis				0.523			
No	60	1					
Yes	123	0.89	0.62–1.27				
Bone metastasis				**0.008**			
No	123	1					
Yes	60	1.59	1.13–2.25				
Liver metastasis				**0.013**			
No	155	1					
Yes	28	1.74	1.12–2.69				
Brain metastasis				**0.009**			
No	169	1					
Yes	14	2.23	1.22–4.07				
Adrenal metastasis				**0.017**			
No	145	1					
Yes	38	1.59	1.08–2.34				
Presence of bone and/or brain and/or liver metastasis				**0.001**			
No	97	1					
Yes	86	1.80	1.28–2.51				
NLR				**<0.001**			
<3.2	106	1					
≥3.2	77	2.12	1.47–3.05				

CI, confidence interval; NLR, neutrophil-to-lymphocyte ratio; μ: The threshold value was determined as the upper limit of normal; ε: The threshold value was determined as the lower limit of normal.

To identify variables associated with PFS following the initiation of first-line therapy, we performed univariate Cox regression analyses on several potential prognostic factors. Our results indicated that anemia, hypercalcemia, low Karnofsky Performance Status, a diagnosis-to-treatment interval of less than 1 year, absence of prior nephrectomy, a greater number of metastatic sites, metastases involving bone, liver, brain, and an elevated NLR were all associated with worse survival outcomes (Table 5). We then conducted a multivariate Cox regression analysis with forward likelihood ratio selection from the full model, and only three independent variables, including anemia, low Karnofsky Performance Status, and presence of bone metastasis, were shown to have independent impacts on PFS (Table 5).

**Table 5:** Univariate and multivariate analysis for progression-free survival.

	n	Univariate analysis			Multivariate analysis		
		HR	%95 CI	HR	%95 CI	HR	%95 CI
Age at diagnosis				0.191			
≤65	143	1					
>65	40	1.27	0.88–1.83				
Gender				0.722			
Male	139	1					
Female	44	0.93	0.65–1.33				
Tumor histopathology				0.332			
Clear cell	147	1					
Non-clear cell	36	1.21	0.81–1.80				
Hemoglobinε				**0.001**			**<0.001**
<12 g/dL	81	1			1		
≥12 g/dL	102	0.45	0.32–0.63		0.46	0.32–0.66	
Neutrophilsμ				0.145			
≤7000/µL	138	1					
>7000/µL	45	1.34	0.90–2.00				
Plateletsμ				0.050			
≤400/10^3^µL	144	1					
>400/10^3^µL	39	1.46	1.00–2.14				
Corrected Calciumμ				**0.003**			
≤10.2 mg/dL	159	1					
>10.2 mg/dL	24	1.91	1.24–2.95				
Karnofsky Performance Status				**<0.001**			**<0.001**
≥80	160	1			1		
<80	23	8.22	4.82–14.03		7.81	4.35–13.99	
Time from diagnosis to treatment				**0.039**			
<1 year	110	1					
≥1 year	73	0.71	0.51–0.98				
Nephrectomy				**0.001**			
No	50	1					
Yes	133	0.56	0.40–0.79				
Metastasis type				0.149			
De novo metastatic	105	1					
Metachronous metastatic	78	0.79	0.57–1.08				
Number of metastases				**0.002**			
<3	118	1					
≥3	65	1.64	1.19–2.27				
Lung metastasis				0.911			
No	60	1					
Yes	123	1.01	0.72–1.42				
Bone metastasis				**0.002**			**0.004**
No	123	1			1		
Yes	60	1.73	1.23–2.42		1.74	1.19–2.53	
Liver metastasis				**0.022**			
No	155	1					
Yes	28	1.64	1.07–2.52				
Brain metastasis				**0.003**			
No	169	1					
Yes	14	2.37	1.35–4.15				
Adrenal metastasis				0.086			
No	145	1					
Yes	38	1.38	0.95–2.01				
Presence of bone and/or brain and/or liver metastasis				**<0.001**			
No	97	1					
Yes	86	2.02	1.46–2.80				
NLR				**<0.001**			
<3.2	106	1					
≥3.2	77	1.90	1.33–2.70				

CI, confidence interval; NLR, neutrophil-to-lymphocyte ratio; μ: The threshold value was determined as the upper limit of normal; ε: The threshold value was determined as the lower limit of normal.

### Meet-URO and IMDC-7 Risk Score

**Figure 1:**
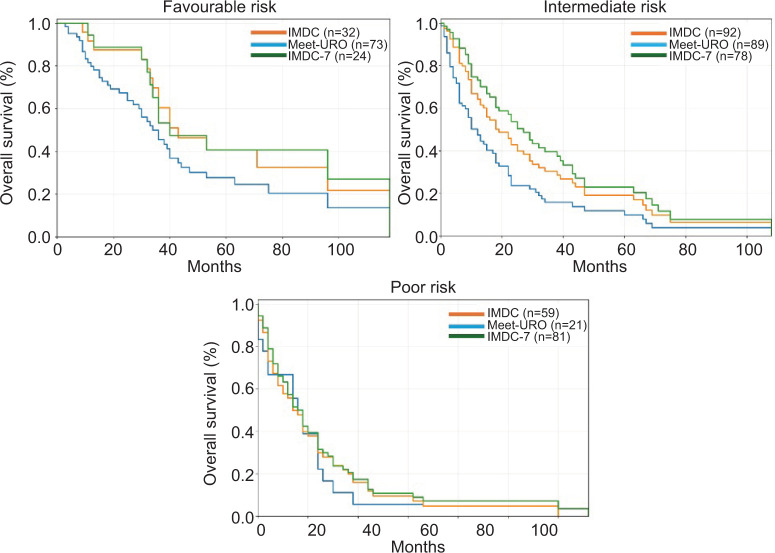
Overall survival within favorable, intermediate, and poor risk groups according to IMDC, Meet-URO, and IMDC-7 scores.

The numerical distribution of patients in each risk category according to the IMDC, Meet-URO, and IMDC-7 scores is presented in Table 6. Overall survival was evaluated separately within the favorable, intermediate, and poor-risk groups across the three prognostic models (Figure 1). When analyzed according to each scoring system, the IMDC score stratified patients into favorable, intermediate, and poor-risk groups, with median OS of 46 (95% CI, 35–56), 22 (95% CI, 16–28), and 7 (95% CI, 4–10) months, respectively (P < 0.001). The Meet-URO score stratified patients into favorable, intermediate, and poor-risk groups, with median OS of 36 months (95% CI, 27–45), 12 months (95% CI, 8–16), and 8 months (95% CI, 5–11), respectively (P < 0.001). The IMDC-7 score similarly stratified patients into favorable, intermediate, and poor-risk groups, with median OS of 40 months (95% CI, 20–59), 27 months (95% CI, 19–35), and 7 months (95% CI, 5–9), respectively (P < 0.001). To assess the prognostic performance of the risk scores, Harrell’s C-indexes for the IMDC, IMDC-7, and Meet-URO scores were 0.655, 0.657, and 0.615, respectively.

**Table 6:** Risk stratification of patients according to IMDC, Meet-URO, and IMDC-7 risk scores.

Variable	All patients (N = 183)
IMDC score, n (%)	
Favorable risk	32
Intermediate risk	92
Poor risk	59
Meet-URO score, n (%)	
Favorable risk	73
Intermediate risk	89
Poor risk	21
IMDC-7 score, n (%)	
Favorable risk	24
Intermediate risk	78
Poor risk	81

## Discussion

This study highlights that the involvement of metastatic sites is closely associated with patient outcomes. Among existing scores, IMDC-7 showed the strongest prognostic performance, underscoring the value of incorporating metastatic site information into risk stratification for TKI-treated patients. Our findings provide real-world evidence from a middle-income country, where IO-based combinations remain less accessible, and TKI monotherapy continues to dominate first-line therapy. In this setting, refined tools such as IMDC-7 are particularly relevant for patient counseling and treatment planning.

Several previous studies have compared prognostic scores in metastatic RCC. In a landmark external validation of over 1000 patients treated with first-line TKI, Heng et al. reported that the IMDC score achieved a c-index of ~0.66 for OS, comparable to the MSKCC score (~0.65–0.66) and to other models (Cleveland Clinic Foundation, IKCWG, French score), which showed c-indices in the ~0.64–0.67 range. These data suggest that scores based solely on routine clinical and laboratory variables tend to show similar, only modestly discriminatory performance (15). Subsequent external validations have reported consistent findings. For example, Tanaka et al. compared the IMDC and MSKCC models in patients receiving first-line versus second-line TKI and observed nearly identical prognostic accuracy (c-index ~0.68–0.69 in the first-line setting, ~0.65–0.66 in the second-line setting) (16). In the era of immune checkpoint inhibitors, the performance of these classic models remains modest. In a long-term analysis of the CheckMate-214 trial (nivolumab + ipilimumab vs sunitinib), Mantia et al. found global c-indices of ~0.66 for IMDC and ~0.64 for MSKCC in the sunitinib-treated patients, and ~0.63 and ~0.61, respectively, in those treated with nivolumab and ipilimumab (17). Collectively, these comparisons highlight that conventional clinical risk scores provide comparable discrimination and underscore the need to incorporate additional factors (e.g., patterns of metastasis or novel biomarkers) to improve prognostic stratification in mRCC.

In our study, the lung was the most frequent site of metastasis, followed by bone, adrenal gland, liver, and brain. These findings are consistent with previous extensive cohort studies and support established patterns in the literature (18, 19). Notably, 47% of our patients had at least one of the following metastases: liver, bone, or brain. This proportion is considerably higher than that reported in the previous study, which found that metastases to these sites were associated with a poor prognosis and limited treatment response (12). The higher frequency in our cohort may reflect a more advanced disease stage at presentation or differences in imaging practices across centers.

Among the metastatic sites, patients with bone, liver, or brain metastases had significantly shorter overall survival compared to those without. This difference was more pronounced when at least one of these sites was involved, consistent with findings from previous studies (11, 12). Bone metastasis was the only site independently associated with progression-free survival, consistent with previous findings that link bone involvement to poor outcomes in mRCC (2, 20, 21). However, no metastatic site showed an independent association with OS in our cohort. In addition to the metastatic site, patients with three or more metastatic sites had significantly worse survival, likely reflecting a higher tumor burden. These findings suggest that both the number and location of metastases should be considered in clinical decision-making.

The Meet-URO score incorporates metastatic sites and systemic inflammation into the IMDC score. It was first developed and validated in patients treated with ICIs (7, 8). Subsequently, it was tested in groups treated with cabozantinib, a TKI, in both first-line and later-line therapies (9, 22). To our knowledge, our study is the first evaluation of the Meet-URO score in patients receiving first-line TKI monotherapy with sunitinib or pazopanib. Although Meet-URO generally performed better than IMDC in studies involving TKIs such as cabozantinib, its prognostic performance was lower in our patient cohort treated with first-line TKIs like sunitinib or pazopanib. The ongoing Meet-URO 33 study (REGAL study) is expected to provide further insight into the prognostic performance of first-line TKIs (23). These differences likely arise from the way the two scores were initially created. IMDC was specifically developed using data from patients treated with TKIs, and extensive research in clinical trials and real-world settings has confirmed its utility. In contrast, the Meet-URO score incorporates factors such as the NLR and bone metastasis, which were tailored initially for ICIs or combination treatments involving ICIs, making it potentially less applicable to TKI-only scenarios. Thus, our findings emphasize the importance of aligning prognostic models with both patient characteristics and specific treatment approaches, supporting the continued use of IMDC in TKI monotherapy settings.

Turning to the IMDC-7 score, it successfully stratified our patients into distinct risk groups, with median OS of 40, 27, and 7 months for the low-, intermediate-, and high-risk categories, respectively. These findings closely align with previous reports in patients treated with targeted therapies or immune checkpoint inhibitors (median OS of approximately 52, 26, and 10 months, respectively) in which external validation also demonstrated a modest improvement in the concordance index compared with the original IMDC score (12). Although metastatic involvement itself is not solely determinative of prognosis, it generally signals an aggressive clinical course. While underlying biological and genomic factors undoubtedly drive metastatic potential, our primary aim is to utilize easily accessible clinical prognostic markers, as genomic and biological markers may not yet be practical for routine clinical use. Therefore, metastatic site involvement remains a promising and clinically feasible parameter for prognostic scores in current clinical practice.

Our study is limited by its retrospective and single-center design. As all patients received TKIs, the results do not represent outcomes associated with current immunotherapy-based combination therapies. Despite this, the study contributes to a better understanding of the prognostic relevance of metastatic pattern, particularly in the context of emerging risk scores. Our results point to the necessity for larger, prospective, and longer-term studies.

## Conclusion

IMDC-7 demonstrated the best prognostic performance among the three models evaluated, slightly outperforming the classical IMDC score. While Meet-URO also provided useful stratification, its overall performance was lower. Our study confirmed the substantial prognostic value of key metastatic sites, including bone, liver, and brain. While previous validations of IMDC-7 and Meet-URO have largely examined ICI-based regimens or later-line TKIs such as cabozantinib, our study focuses specifically on first-line sunitinib and pazopanib, addressing an important evidence gap in an ICI-free setting. To our knowledge, this is among the first real-world evaluations of these models in patients receiving TKI monotherapy. These findings support the use of extended models, such as IMDC-7, to guide more accurate risk assessment in daily clinical practice.
